# Improving the Storage Stability of Tomato Paste Enriched by Different Concentrations of Zinc Micronutrient

**DOI:** 10.1002/fsn3.71106

**Published:** 2025-10-21

**Authors:** Rashid Gholami, Nahid Aghilinategh

**Affiliations:** ^1^ Department of Agricultural Machinery Engineering, Sonqor Agriculture Faculty Razi University Kermanshah Iran

**Keywords:** electronic nose, storage stability, tomato paste fortification, zinc

## Abstract

This study evaluates the fortification of tomato paste with zinc (Zn) micronutrient and its impact on quality control during storage. Industrial tomato paste was divided into four groups: a control group and three groups fortified with 20 ppm zinc (Zn‐20 ppm), 40 ppm zinc (Zn‐40 ppm), and 60 ppm zinc (Zn‐60 ppm). The samples were stored at 4°C for 60 days, with physical tests: L*, a*, b*, ∆*E*, and a*/b*, chemical tests: pH, total soluble solids (TSS), lycopene, total phenol content (TPC), and antioxidant capacity (AC), and microbiological tests: acid‐resistant mesophilic bacteria (ARMB) and mold and yeast (MY), assessed every 15 days. Additionally, the aroma properties of the paste were analyzed using an electronic nose system. The results demonstrated a decline in L*, a*, b*, and a*/b* indices and reductions in lycopene, TPC, and AC levels across all treatments during storage. In contrast, ∆*E*, ARMB, and MY counts increased over time. Lycopene, TPC, and AC exhibited the highest reductions in the control group by the end of storage, at 84.72%, 16.29%, and 38.69%, respectively, while the Zn‐40 ppm group showed the minor decreases, with reductions of 70.85% for lycopene, 3.39% for TPC, and 22.86% for AC. Analysis of variance (ANOVA) indicated that the storage period, fortification levels, and their interactions had significant effects (*p* ≤ 0.01) on the properties of the tomato paste. The overall accuracy of quadratic discriminant analysis (QDA) in identifying and distinguishing samples across all storage periods was 100%, with no misclassifications among the 32 samples analyzed.

## Graphical abstract

In this research, tomato paste as a high consumption food product has been enriched with zinc micronutrient using certain concentrations. Some of the main results were: Zinc fortification of tomato paste enhances its nutritional value. Zinc‐fortified samples showed less degradation of total phenol content (TPC) and antioxidant capacity (AC). The results of the electronic nose system showed that fortification with Zinc had no significant effect on the smell of tomato paste. Zinc fortification proved beneficial in maintaining product quality over the 60‐day storage period.

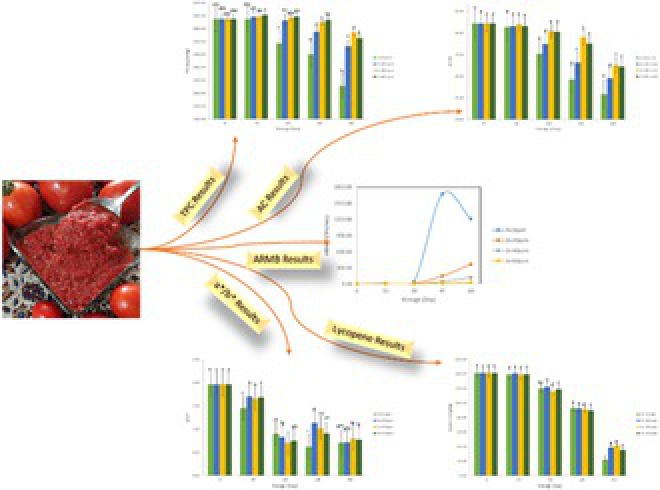

## 1 Introduction

Tomatoes are consumed in their raw form and processed into various products such as paste, puree, and juice. According to reports from the Food and Agriculture Organization (FAO), Iran produces approximately 3,400,000 tons of tomatoes annually (Anonymous 2023), with over 600,000 tons processed into tomato paste. Beyond their taste, flavor, and aroma, tomato paste is widely consumed in numerous countries, including Iran, due to its rich nutritional content and natural antioxidant properties. Both epidemiological and clinical studies have underscored the substantial health benefits of tomatoes and their derived products. Tomatoes are an excellent source of essential micronutrients, including β‐carotene (as precursor to vitamin A), vitamin C, folate, and potassium. They also contain bioactive compounds like lycopene and polyphenols, recognized for their strong antioxidant potential. Lycopene, the most abundant carotenoid in tomatoes, possesses a highly conjugated molecular structure that helps reduce oxidative stress. Its ability to quench singlet oxygen is twice that of β‐carotene (Giovannucci 2002; Lavelli et al. 2000; Sesso et al. 2003). Poor dietary habits, such as low fruit and vegetable consumption, are known risk factors for numerous health issues and diseases. Extensive epidemiological research has shown that cardiovascular health is significantly influenced by a nutrient‐rich diet (Cheng et al. 2017). The relationship between specific foods and their health benefits has been the subject of scientific investigation for many years. As a result, the development of functional foods that promote health and wellness has become a priority in the food industry. This trend has led to increased consumption of fortified foods containing physiologically active components, such as prebiotics, probiotics, vitamins, minerals, dietary fiber, fish oil, and plant sterols (Betoret et al. 2003). Fortification has become a key strategy to address nutritional deficiencies in response to growing consumer demand for high‐quality, health‐promoting foods. Nutrition experts regard food fortification as one of the most effective methods to provide essential micronutrients. It involves adding one or more vital nutrients to foods at levels higher than those naturally present to prevent or correct nutrient deficiencies in the population or specific demographic groups (Stanton et al. 2001). Despite the growing interest in food fortification, research on enhancing processed tomato products remains relatively limited. Some examples of current fortification efforts include: the addition of tomato seed oil to tomato juice (Ghasemi Baghabrishami and Goli 2023), enrichment of tomato sauce and diced tomatoes with tomato components (Tagliamonte et al. 2023), improvement of tomato paste shelf life by incorporating encapsulated olive leaf (Jafari et al. 2021), fortification of tomato paste with 6% tomato peel (Reboul et al. 2005), and rosemary essential oil microemulsions as antimicrobial and antioxidant agents in tomato paste (Brandt et al. 2023).

Zinc (Zn) is an essential mineral required for numerous biological processes in the human body. The recommended daily intake (RDI) of zinc is 8 mg for adult women and 11 mg for adult men, which is better to provide only through diet. The zinc content in breast milk is high during the first 2 weeks postpartum but decreases significantly throughout lactation (Krebs et al. 1995). Supplementing or fortifying foods and beverages with zinc must meet the body's requirements. Tomato paste is one of the most widely produced industrial tomato products and is a critical condiment in modern diets. Given its widespread consumption, fortifying tomato paste with zinc compounds has the potential to create a functional food product.

In addition to evaluating food products' physical, chemical, and microbiological properties, the electronic nose system is a nondestructive method increasingly used to assess the quality of agricultural and food products. This system mimics the human sense of smell and utilizes sensors to detect odors in the headspace of samples (Modupalli et al. 2021). Several studies have explored the application of electronic nose technology in the food industry (Sanaeifar et al. 2017).

However, fortifying tomato paste with zinc for quality improvement during storage has not been thoroughly investigated. Given the rising incidence of zinc deficiency‐related health issues and the gap in research on this topic, the present study aims to fortify tomato paste with varying concentrations of zinc compounds (0, 20, 40, and 60 ppm). In addition to fortification, the effects of different zinc concentrations on the aromatic, physical, mechanical, microbiological, and chemical properties of tomato paste were evaluated throughout the storage period.

## 2 Materials and Methods

### 2.1 Sample Preparation

Tomato paste samples with specific characteristics (TSS = 27.9 and pH = 4.15) were obtained on the day of production from *Sahar Food Industries*, located in Hamadan Province, Iran. On the first day, the samples were transferred under controlled conditions (at 4°C) to the *Pishgaman‐e‐Part laboratory*, where the fortification process was conducted. The samples were divided into a control group and those fortified with 20, 40, and 60 ppm of Zinc. Initially, each tomato paste sample was subdivided into three 9.98, 9.96, and 9.94 g portions, and 20, 40, and 60 mg of Zinc metal were added to each respective portion. These portions were then thoroughly mixed with 990 g of tomato paste, resulting in final concentrations of 20, 40, and 60 mg per kilogram (ppm). Following classification, the fortified tomato paste samples were packed in designated containers and stored for 60 days at 4°C with 75% relative humidity. Tests were conducted regularly, specifically on days 15, 30, 45, and 60 for all treatments.

### 2.2 Physicochemical Properties

#### 2.2.1 Total Phenol Content (TPC), Lycopene, and Antioxidant Capacity (AC)

The Folin–Ciocalteu method was employed to measure the TPC. Initially, 5 g of tomato paste was extracted with 3 mL of 85% methanol. Subsequently, 300 μL of the extract was added to 1500 μL of 10% Folin–Ciocalteu reagent and left in the laboratory environment for 5 min. Then, 1200 μL of 7% sodium carbonate (Na_2_CO_3_) and 600 μL of distilled water (H_2_O) were added, and the mixture was shaken at 110 rpm at room temperature for 90 min. Finally, the sample absorbance was measured at a wavelength of 765 nm using a spectrophotometer (Unico 2100 UV–Vis) and reported as milligrams of gallic acid per gram of sample tissue, based on comparison with a gallic acid standard (Singleton and Rossi Jr. 1965; Katırcı et al. 2020).

Lycopene is the most abundant carotenoid in tomato products, with β‐carotene and lutein also present in the products and their bioaccessible fractions (Tagliamonte et al. 2023). An extraction solution was prepared to measure the lycopene concentration by combining 5 mL of 95% ethanol, 5 mL of pure acetone, and 10 mL of hexane. A tomato paste sample weighing between 0.4 and 0.6 g was added to this solution in a container. The mixture was shaken at 180 rpm for 15 min to achieve homogeneity. Subsequently, 3 mL of distilled water was added, and the sample was shaken for 5 min under the same conditions. The mixture was then allowed to rest at room temperature for 5 min to facilitate phase separation. The hexane phase (the upper layer) was carefully extracted, and the sample's absorbance was measured at 503 nm using a UV–Vis spectrophotometer (Unico 2100) (Fish et al. 2002).

The AC of the samples was assessed based on their ability to scavenge free radicals using the DPPH (2,2‐diphenyl‐1‐picrylhydrazyl) radical scavenging capacity method, calculated as a percentage (%). In this method, 500 μL of the extract (0.5 g of the sample extracted with 3 mL of 85% methanol) was prepared and mixed with 500 μL of distilled water for 5 min, followed by centrifugation at 1000 rotations per minute for 5 min. Subsequently, 75 μL of the obtained solution was transferred to test tubes, and finally, 2925 μL of DPPH methanolic solution (with a concentration of 2.4 mg per 100 mL of 85% methanol) was added. After a few seconds, the absorbance of the sample solutions and the control were measured at a wavelength of 515 nm using a spectrophotometer (Unico 2100 UV–Vis). Finally, the Radical Scavenging Capacity (RSC) percentage was calculated using the following formula (Brand‐Williams et al. 1995) Article 3:

$$\mathrm{RSC}\;\left(\%\right)=\left(\frac{A_{1}-A_{2}}{A_{1}}\right)\times 100$$

where *A*_1_ and *A*_2_ are control and sample absorption, respectively.

#### 2.2.2 pH and Total Soluble Solids (TSS)

The chemical properties of food products, such as tomato paste, play a crucial role in determining their quality during production and storage, as they provide indirect insights into internal changes, including microbial activity (Ganje et al. 2016). These chemical properties include TSS and pH. This study measured the chemical properties in triplicate at each evaluation period. TSS was measured with an Atago refractometer model PLA‐2 from Japan with a resolution of 0.01 at 25°C. Before measuring, the refractometer was calibrated with distilled water, after which the uniform tomato paste samples were filtered through filter paper, and a few drops were used to obtain the TSS value.

#### 2.2.3 Color Indices

Color is one of the most important physical factors in food products, as it directly and immediately impacts the product's quality and marketability. Primary color indices such as L* (lightness), a* (redness), and b* (yellowness), as well as secondary indices such as color difference (∆E), play a critical role in determining the appearance quality of agricultural products during storage. In the case of tomato paste, the a*/b* ratio is also a key indicator of quality (Elbadrawy and Sello 2016). In this study, the primary indices were measured using a colorimeter (model HP‐200 from Shenzhen Handsome Technology Co. Ltd.), and secondary indices were calculated using the following formulas (Gholami et al. 2020):

$$∆E=\sqrt{∆L^{*2}+∆a^{*2}+∆b^{*2}}$$

### 2.3 Microbiological Tests

The mold and yeast (MY) in the tomato paste samples were assessed using the Howard mold count method (AOAC 965.41, 1965). To evaluate the presence of acid‐resistant mesophilic bacteria (ARMB), a pour plate culture was prepared using a 0.1% dilution in sterile physiological saline, and the plates were incubated in Plate Count Agar (PCA) at 30°C for 72–120 h. For the total microorganism count, a pour plate culture was prepared using a 0.1% dilution in sterile physiological saline, and the plates were incubated in Yeast Glucose Chloramphenicol (YGC) medium at 25°C for 48–72 h (Njongmeta et al. 2013).

### 2.4 Mechanical Testing

The viscosity and consistency of the fortified and control tomato paste samples were measured using the Bostwick method. This method measures the flow of a specific fluid volume under its weight over a fixed period and is a reliable indicator of viscosity, though it is less applicable to very thick materials (Adekunte et al. 2010). To assess consistency, the tomato paste samples were diluted with distilled water to 12 Brix and tested at 25°C using a Bostwick viscometer. The results were recorded as the distance traveled in centimeters over 30 s.

### 2.5 Electronic Nose System

The electronic nose system used in this study was detailed in an investigation conducted by Gholami et al. (2023).

### 2.6 Data Analysis

The experimental data were analyzed using SPSS version 19 software for ANOVA (Analysis of Variance) and Duncan's multiple range test to compare means. Additionally, predictive models for the measured variables were generated using Design Expert software, applying the quadratic model (QM). Models with a predicted R‐square value greater than 0.85 for each characteristic were presented in the results. The QM for each factor was expressed as follows:

$$\alpha =\beta_{0}+\beta_{1}A+\beta_{2}B+\beta_{3}\mathrm{AB}+\beta_{4}A^{2}+\beta_{5}B^{2}$$

where: *α* represents the measured characteristic, *β*_0_ is the constant term, *β*_i_ are the coefficients, *A* is the storage period, and *B* is the zinc concentration.

In addition, the preprocessed data were analyzed using multivariate discriminant analysis (QDA) and Principal Component Analysis (PCA) with Unscrambler version 9.7 and Matlab 2015a software. PCA is a statistical method that converts correlated data sets into uncorrelated variables, known as principal components (Esteki et al. 2017). QDA is a statistical technique used to identify the linear and quadratic combinations of characteristics that best differentiate between two or more groups of objects (Chapman et al. 2001). The accuracy and performance of the models were evaluated using sensitivity, specificity, and precision indices, as outlined in the authors' previous work (Gholami et al. 2023).

## 3 Results and Discussion

### 3.1 TPC, AC and Lycopene

**FIGURE 1:**
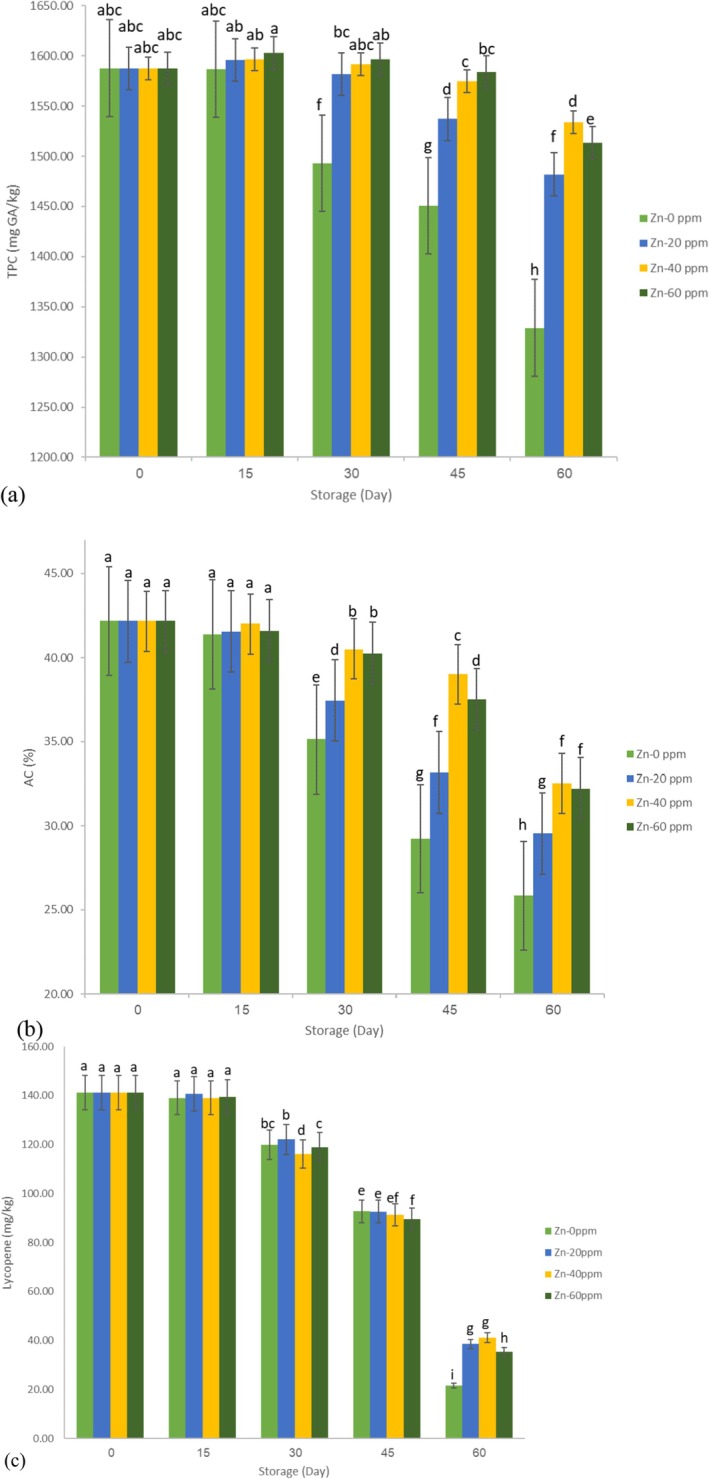
Changes in (a) TPC, (b) AC, and (c) lycopene during the storage period.

Figure 1 shows the changes in TPC, AC, and lycopene levels over the storage period. At the beginning of the storage period, the TPC, AC, and lycopene content were 1578.64 mg GA/kg, 42.17%, and 141.13 mg/kg, respectively. The results indicated a steady decline in all three components, with the reduction becoming more pronounced after the first 15 days of storage. Researchers have reported similar findings and observed significant decreases in lycopene and some other ingredients while storing tomatoes and tomato‐based products (Lin and Chen 2005). By the end of the storage period, the most significant reductions in lycopene (84.72%), TPC (16.29%), and AC (38.69%) were recorded in the control sample. In contrast, minor reductions were 70.85%, 3.39%, and 22.86%, respectively, which were observed in the Zn‐40 ppm sample. The ANOVA results (Table 1) indicated that the storage period, zinc fortification, and their interaction had a significant effect (*p* < 0.01) on the changes in lycopene, TPC, and AC. Duncan's comparison of means test (Table 2) revealed that lycopene and TCD changes between days 0 and 15 were not statistically significant, whereas significant differences were observed between the other time points. Additionally, changes in AC were significant across all storage days. The change in zinc concentration from 40 ppm to 60 ppm did not significantly affect lycopene and TPC levels, but there were significant differences between this range and other concentrations. Zinc concentrations significantly affected AC levels at all tested concentrations. The results of compare means test of interactions effect (storage × Zinc concentration) are shown as letters on Figure 1, which similar letters show insignificant effects. Overall, the results demonstrated that the samples fortified with zinc experienced less degradation in lycopene, TPC, and AC by the end of the storage period. This outcome could be attributed to the role of zinc in stabilizing tomato paste, as it may help limit enzymatic activity and reduce oxidation, a conclusion supported by other studies as well (Ordóñez‐Santos et al. 2009). Furthermore, research has indicated that using food additives in tomato paste can enhance its antioxidant activity (Mezza et al. 2018), which could also be relevant to this study. Lycopene content is directly related to the color of tomatoes and tomato products, and it was observed that Zn fortification helped control both color changes and lycopene levels during storage. Given the significant effects of both storage time and fortification, the following predictive relationships for TPC, AC, and lycopene were derived using the QM, with correlation coefficients of 0.90, 0.96, and 0.97, respectively:

$$\mathrm{TPC}=1591.31-62.42A+41.34B+39.83\mathrm{AB}-38.82A^{2}-40.09B^{2}\;R^{2}=0.90$$

$$\mathrm{AC}=39.32-6.53A+1.67B+0.91\mathrm{AB}-2.82A^{2}-1.55B^{2}\;R^{2}=0.96$$

$$\text{Lycopene}=119.95-53.63A+1.97B+2.58\mathrm{AB}-30.53A^{2}-2.96B^{2}\;R^{2}=0.97$$

**TABLE 1:** ANOVA analysis of the properties of tomato paste during the storage period.

	a*	a*/b*	∆E	Viscosity
Model	234.578**	90.356**	85.019**	218.713**
Day	894.954**	336.339**	139.281**	833.703**
Zn%	11.037**	6.935**	193.147**	50.824*
Day×Zn	12.430**	9.690**	52.262**	13.899**

	pH	TSS	TPC	AC
Model	128.408**	67.785**	154.855**	332.747**
Day	263.489**	258.239**	367.592**	1132.604**
Zn%	19.371**	6.918**	316.925**	284.739**
Day×Zn	70.520**	4.958**	38.940**	36.972**

	Lycopene	ARMB	MY
Model	1833.992**	879.072**	621.552**
Day	7246.505**	1177.503**	1220.951**
Zn%	20.030**	1790.067**	822.730**
Day×Zn	23.744**	606.858**	369.575**

*Note:* *Significant at 5% level; **Significant at 1% level.

Abbreviation: ns, not significant.

**TABLE 2:** Duncan's comparison of means results.

		a*	a*/b*	∆E	Viscosity
Day	0	9.72^a^	1.96^a^	0^d^	6.00^a^
	15	7.65^b^	1.62^b^	1.61^b^	5.96^a^
	30	2.92^c^	0.79^d^	2.54^a^	5.85^b^
	45	2.89^c^	0.91^c^	1.17^c^	5.63^c^
	60	2.86^d^	0.71^d^	1.21^c^	4.87^d^
Zn	0	4.77^a^	1.12^a^	2.59^a^	5.53^b^
	20	3.90^c^	1.05^b^	1.23^b^	5.63^a^
	40	4.01^bc^	1.04^b^	1.02^c^	5.63^a^
	60	4.15^bc^	1.02^b^	1.05^c^	5.64^a^

		pH	TSS	TPC	AC
Day	0	4.15^a^	27.80^a^	1587.64^a^	42.16^a^
	15	4.04^c^	27.22^b^	1595.68^a^	41.63^b^
	30	4.01^d^	27.13^b^	1565.71^b^	38.34^c^
	45	4.04^c^	25.92^c^	1536.59^c^	34.73^d^
	60	4.05^b^	25.73^d^	1464.47^d^	29.58^e^
Zn%	0	4.07^a^	26.63^a^	1489.37^c^	34.75^d^
	20	4.04^b^	26.50^a^	1549.24^b^	35.43^c^
	40	4.03^b^	26.64^a^	1574.17^a^	37.44^b^
	60	4.03^b^	26.52^a^	1574.25^a^	38.51^a^

		Lycopene	ARMB	MY
Day	0	141.13^a^	0.00^c^	0.00^c^
	15	139.53^a^	3.33^c^	0.00^c^
	30	119.24^b^	14.16^c^	2.50^c^
	45	91.54^c^	470.00^a^	19.16^b^
	60	34.15^d^	434.16^b^	87.16^a^
Zn	0	102.88^a^	588.66^a^	59.06^a^
	20	98.45^b^	131.66^b^	22.50^b^
	40	96.86^c^	42.50^c^	8.33^c^
	60	95.82^c^	11.66^d^	4.16^d^

*Note:* Same letters show no significant effects.

As indicated by these relationships, the storage period had a negative effect (leading to reductions), while fortification positively influenced all three factors.

### 3.2 pH and TSS

**FIGURE 2:**
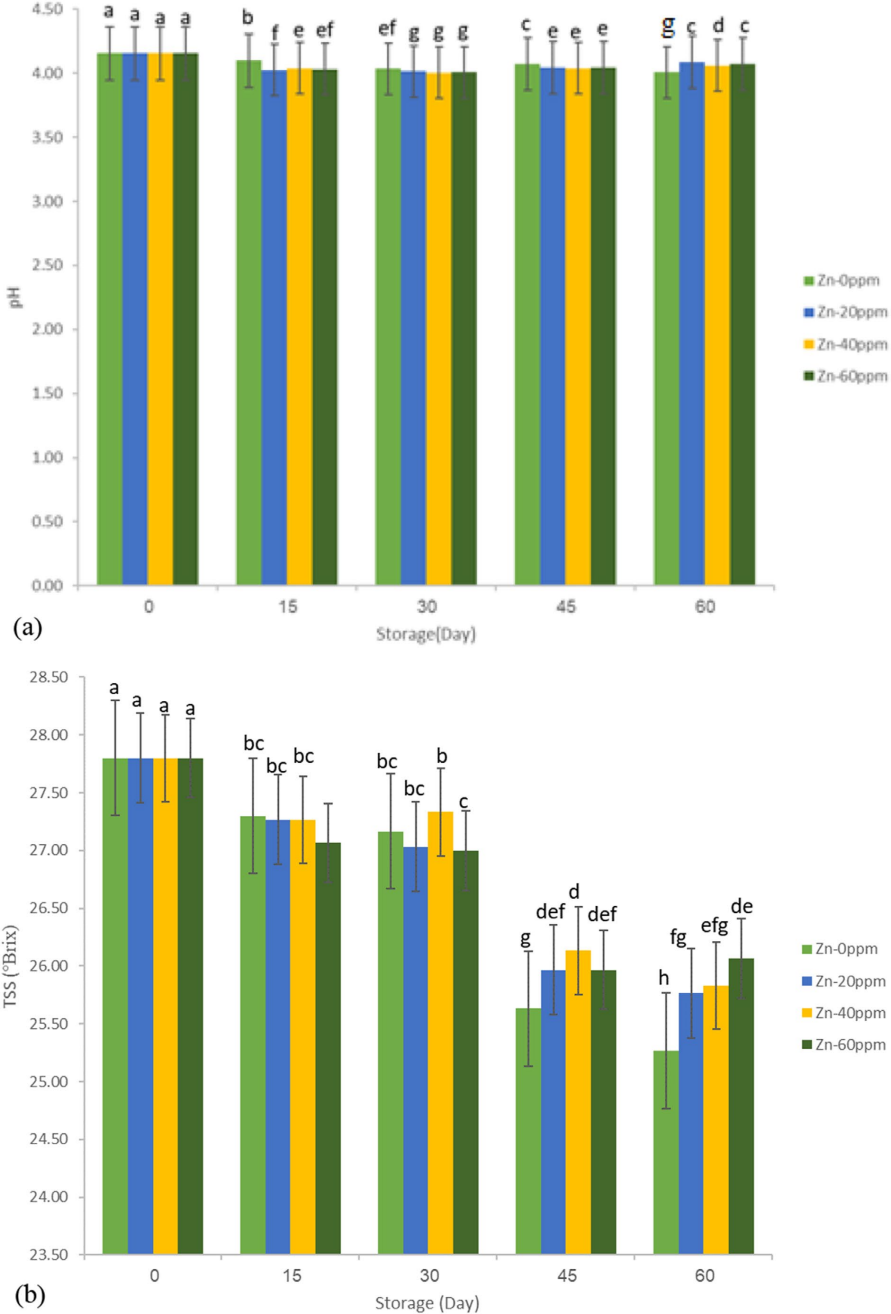
Shows the changes in chemical indices: (a) pH and (b) TSS.

The initial analysis showed that the pH of the tomato paste sample was 4.15, which is generally below the typical market range of 4.4 for tomato paste products. Some studies have reported pH values between 4.07 and 4.23 (Anthon et al. 2011; Katırcı et al. 2020). Throughout the storage period, the pH decreased across all treatments (Figure 2), with the most significant decrease seen in the control sample, which dropped to 4.01 (a 3.53% reduction from the initial value), and the minor decrease in the Zn‐20 ppm sample, which dropped to 4.08 (a 1.69% reduction). This suggests that the use of zinc micronutrient can play a similar role as synthetic preservatives in stabilizing the pH index, which can reflect internal changes in tomato paste during storage. Due to the high concentration of acids and low pH in agricultural and food products such as tomato paste, these products are self‐defense from microbial spoilage. Therefore, only organisms that are able to grow in such conditions can cause spoilage. As noted in previous studies, pH showed minimal changes during storage (Brandt et al. 2023; Roy et al. 2024). The pH change may be attributed to differences in metabolic processes. A reduction in pH values can be linked to increased TA of tomato products, possibly resulting from decreased respiration rates in fresh products (Roy et al. 2024).

On day one, the tomato paste's initial TSS value was 27.8°Brix. During the storage period, TSS values decreased in all treatments. A reduction in TSS can lead to increased fluidity in tomato paste (Jafari et al. 2021), likely due to a decrease in soluble solids or dilution of the solution. The viscosity results corroborated this. The most significant change in TSS occurred in the control sample, with a 9.11% reduction, while the most minor change was observed in the Zn‐60 ppm sample, with a 6.24% reduction. Overall, these results suggest that zinc concentration played an essential role in maintaining the quality of the product during storage, as the least change in chemical indices was observed in the fortified treatments. Other studies have also shown the positive effect of food additives on product stability during storage (Ghasemi Baghabrishami and Goli 2023; Jafari et al. 2021). The ANOVA results (Table 1) further confirmed that fortification, storage period, and their interaction had significant effects (*p* ≤ 0.01) on the changes in both indices. On the other hand, the results of the compare means test of interactions effect (storage × Zinc concentration) are shown as letters on Figure 2, which similar letters show insignificant effects. Finally, predictive equations for the chemical indices were developed using the QM based on the treatments. Models with high correlation coefficients (above 0.85) are presented below:

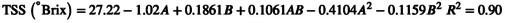




The correlation coefficients for the pH prediction models were 0.74.

### 3.3 Color Indices

**FIGURE 3:**
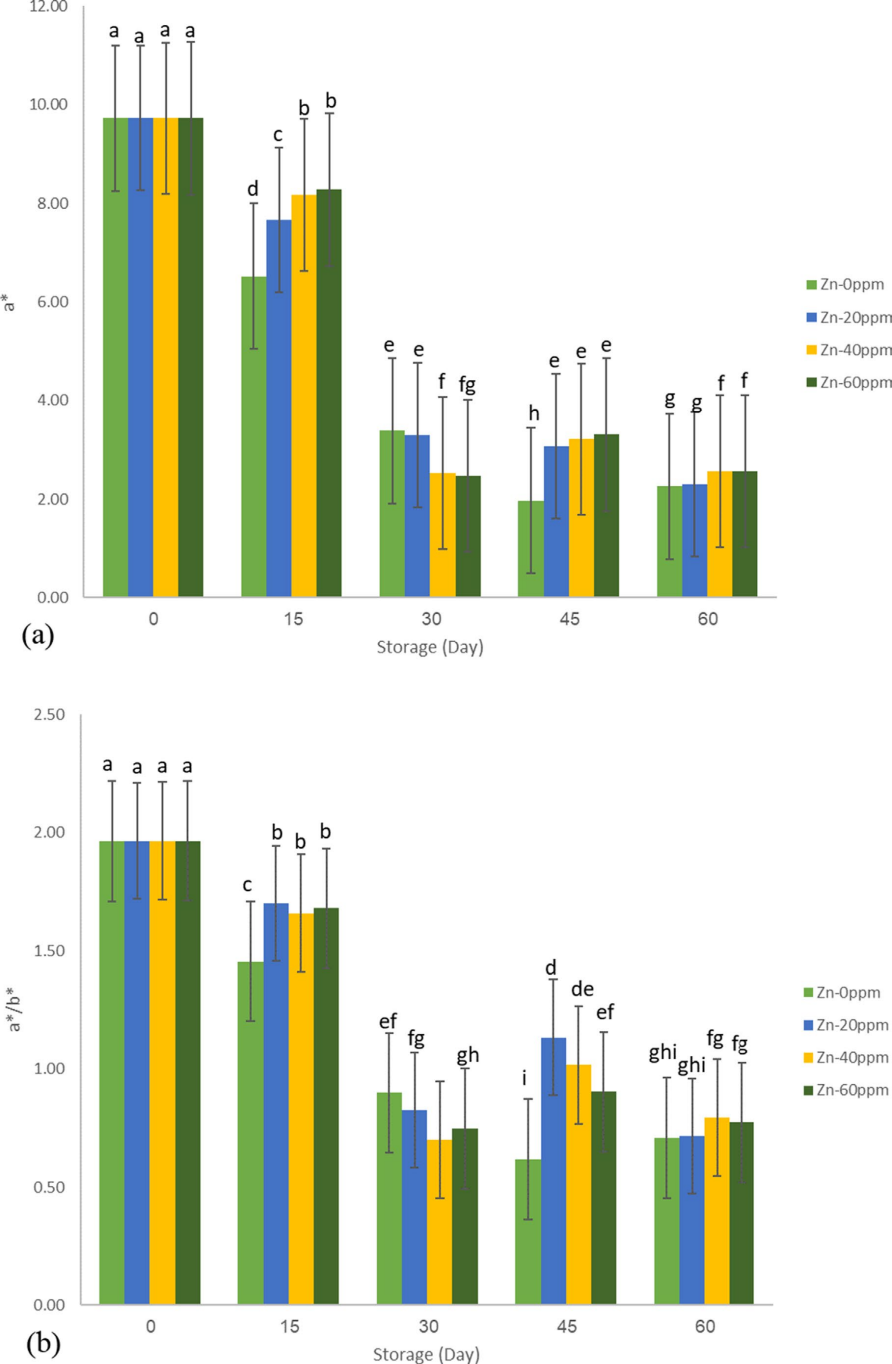
The changes in color indices (a) a* and (b) a*/b*, throughout the storage period.

Evaluating color indices is critical for all agricultural products, as they significantly impact marketability and consumer preference. In this study, the primary color indices of tomato paste were assessed throughout the storage period. The characteristic color of tomato‐based products, like tomato paste, combines red and yellow hues, primarily due to lycopene, lutein, and the total carotenoid content. Consequently, Hunter a* and b* values, or their combination, serve as essential physical parameters for evaluating visual color degradation (Adekunte et al. 2010). In this research, the lightness index (L*), red‐green index (a*), and yellow‐blue index (b*) were measured during storage. The a*/b* ratio, along with color change (∆*E*), was calculated using the formulas outlined earlier. Positive a* and b* values at the beginning and throughout storage confirmed the dominance of red and yellow colors in the tomato paste. The red color mainly results from carotenoids, particularly lycopene, abundant in tomato paste (Ghasemi Baghabrishami and Goli 2023). The a*/b* ratio is a crucial factor in determining tomato paste's color quality, with values of 2 and above indicating superior color and between 1.8 and 2 is acceptable (Katırcı et al. 2020). At the start of the storage period, the control sample's L*, a*, and b* values were 13.85, 9.72, and 4.75, respectively. Additionally, the a*/b* ratio (Figure 3) was 1.96, indicating acceptable initial tomato paste quality based on previous research. During storage, a downward trend was observed in all samples (including those fortified with different zinc concentrations and the control) for all primary color indices (L*, a*, b*, and a*/b*). This observation aligns with findings from other studies on the color indices of tomato paste (Jafari et al. 2021). In contrast, the ∆*E* showed an increasing trend. By the end of the 60‐day storage period, the Zn‐40 ppm fortified sample exhibited the highest L* value (7.10), indicating the least change, while the control sample showed the lowest L* value (6.92), indicating the most change. Additionally, the Zn‐60 ppm fortified samples had the highest a* and b* values (2.57 and 3.32, respectively), reflecting the slightest color change, while the control samples had the lowest values for a* and b* (2.26 and 3.20, respectively). ANOVA results indicated that the storage period significantly affected all three color indices (L*, a*, b*) at the 99% confidence level, whereas Zinc fortification had an insignificant effect on the L* value (Table 1). Duncan's multiple range test (Table 2) revealed significant differences between various Zinc concentrations. Initially, the addition of Zinc at any concentration resulted in a decline in the color indices of the paste. However, the Zinc additive throughout storage contributed to maintaining color stability and controlling the indices compared to the control sample. A study by Jafari et al. (2021) on improving the stability of tomato paste using encapsulated olive leaf also demonstrated that additives positively affected color preservation during storage. On the other hand, as long as the pH index is changing, the color quality will be decreasing, which is attributed to the instability of pigments in tomato paste with pH fluctuations. Furthermore, analysis of the ∆E showed that the storage period significantly influenced this parameter, while Zinc concentration significantly affected color change at the 99% confidence level. On the other hand, the results of the compare means test of interactions effect (storage × Zinc concentration) are shown as letters on Figure 3, which similar letters show insignificant effects. Predictive models for the color indices were developed using the Quadratic model in the Design Expert software based on the significant impact of treatments on the appearance of the tomato paste. The models with a high correlation coefficient (85 or above) are presented below:

$$L^{*}=9.30-3.35A-0.1326B-0.2858\mathrm{AB}+1.08A^{2}+0.0184B^{2}\;R^{2}=0.86$$

$$a^{*}=2.61-3.37A-0.0746B+0.1107\mathrm{AB}+3.21A^{2}+0.3397B^{2}\;R^{2}=0.97$$

$$b^{*}=3.80-0.8867A-0.0481B+0.0342\mathrm{AB}+0.3327A^{2}-0.0886B^{2}\;R^{2}=0.88$$

As illustrated in the models, the storage period and zinc fortification reduced the color indices, resulting in a negative impact. However, as previously mentioned, zinc fortification helped mitigate the reduction of color indices during the storage period.

### 3.4 Acid‐Resistant Mesophilic Bacteria (ARMB), Mold and Yeast (MY)

**FIGURE 4:**
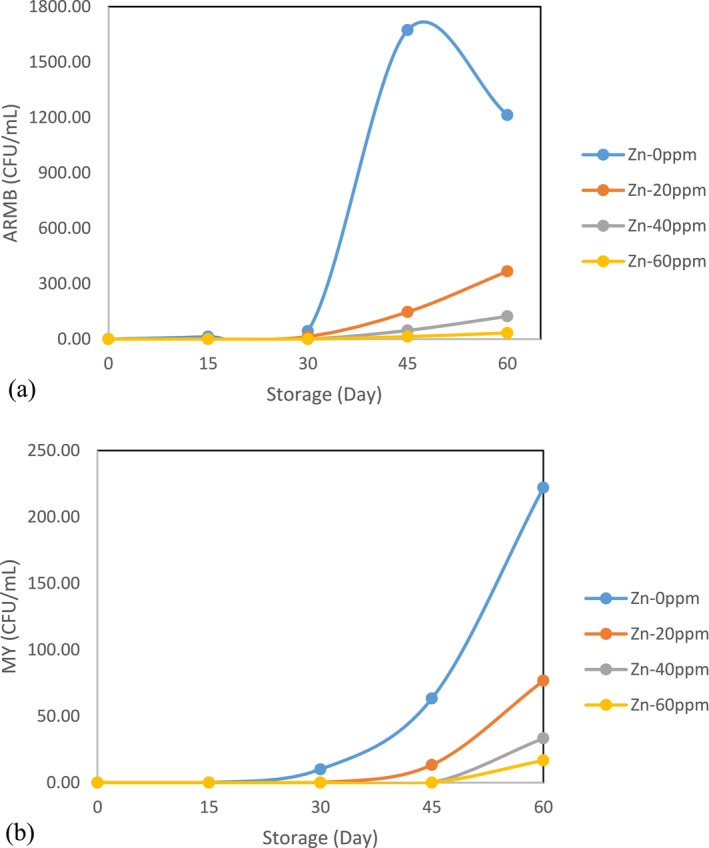
Changes (a) ARMB, and (b) MY during the storage period.

At the start of the experiment, the levels of ARMB and MY were measured. The initial values in the control sample were 0 for both. Over time, a sharp increase in these factors was observed in the control samples after day 30 and all treatments after day 45. By the end of the storage period, the highest levels of ARMB (1213.33 CFU/mL) and MY (222 CFU/mL) were found in the control sample. The control of these increases during the first 30 days in all treatments is likely due to the thermal processing applied during tomato paste production, which maintained the stability of the paste in the initial stages of storage. The minor increases in ARMB and MY were recorded in the Zn‐60 ppm sample by the end of the storage period, with values of 33.33 CFU/mL and 16.67 CFU/mL, respectively (Figure 4). The presence of zinc in the fortified tomato paste enriched the product and contributed to its stability during storage by inhibiting the growth of these microorganisms. The reason for controlling the microbial levels in enriched tomato paste could be that zinc acts as an antimicrobial agent and may have prevented the growth and development of bacteria by destroying the cell wall and cell membrane. Since gram‐positive bacteria have cell walls with teichoic acids and a higher number of peptidoglycan layers, they provide thicker and stiffer walls, and therefore higher concentrations of the additive will be required to limit their growth. Other studies have similarly demonstrated that certain additives, such as rosemary essential oil microemulsions, can effectively control the growth of gram‐positive and gram‐negative bacteria while storing tomato paste. Their results showed that a higher concentration of the additive was needed to control gram‐positive bacteria (Brandt et al. 2023). In the other research on tomato paste enriched with different iron concentrations, it was reported that the increase of acid‐resistant thermophilic bacteria, microorganisms, and mold spores were controlled in enriched samples during storage (Aghilinategh et al. 2025), which confirmed our results about fortification. The ANOVA results (Table 1) showed that the storage period, zinc fortification, and their interaction had significant effects (*p* < 0.01) on the changes in both factors. Duncan's comparison of means test revealed that changes in ARMB and MY were not significant until day 30, but significant differences were observed on subsequent days. Significant differences were also observed between the control and fortified samples and among the different fortification concentrations for both factors. Given the significant effect of the treatments on changes in ARMB and MY, correlation coefficients of 0.50 and 0.62, respectively.

### 3.5 Mechanical Properties—Viscosity

Viscosity is a critical factor in determining the characteristics of products such as tomato paste. At the start of the study, the initial viscosity of the tomato paste was measured at 6 cm. Throughout the storage period, a decline in viscosity was observed across all treatments. By the end of the storage period, the viscosity values for the control, Zn‐20 ppm, Zn‐40 ppm, and Zn‐60 ppm samples were recorded as 4.57, 4.93, 5.03, and 4.97 cm, respectively. The most significant decrease in viscosity occurred in the control sample, with a reduction of 23.89%, while the Zn‐40 ppm sample experienced the most minor reduction at 16.11%. The reason why the viscosity changes in the enriched samples were less than that of the control sample can be attributed to the effect of the presence of the additive as a thickener on the mechanical properties of the paste. The additive also controls the enzymatic activity and ultimately maintains the mechanical nature of the paste during storage. The results from the ANOVA indicated that the storage period significantly affected viscosity at the 99% confidence level, and fortification had a significant effect at the 95% confidence level. Furthermore, a comparison of means revealed no significant difference in viscosity between day zero and day 15; however, significant differences were noted on subsequent storage days. In contrast, there was no significant difference in viscosity among the various zinc concentrations; a significant difference was observed between the fortified samples and the control. The correlation coefficient between the predicted values from the Quadratic model and the experimental values was 0.93. The prediction equation for viscosity is presented below:

$$\mathrm{Vis}=5.89-0.5665A+0.1215B+0.0869\mathrm{AB}-0.3871A^{2}-0.1187B^{2}\;R^{2}=0.93$$

The negative coefficient for the storage period in the model indicates its reducing effect on viscosity, while the positive coefficient for zinc fortification demonstrates its increasing effect, consistent with the percentage changes discussed earlier.

**FIGURE 5:**
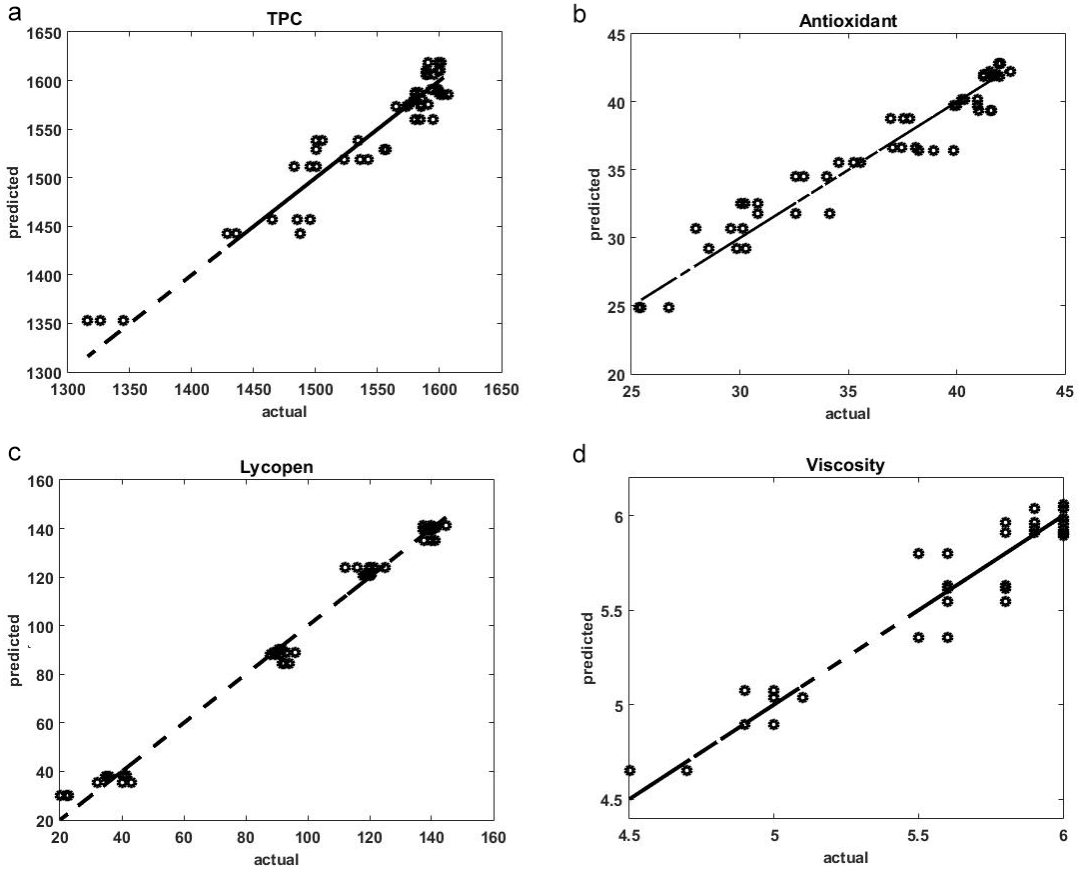
Actual data vs. predicted data for (a) TPC, (b) AC, (c) Lycopene, and (d) viscosity.

Finally, as an example, a plot of actual data versus predicted data using the model equations is presented in Figure 5 for TPC, AC, Lycopene, and viscosity.

### 3.6 Electronic Nose Analysis

**FIGURE 6:**
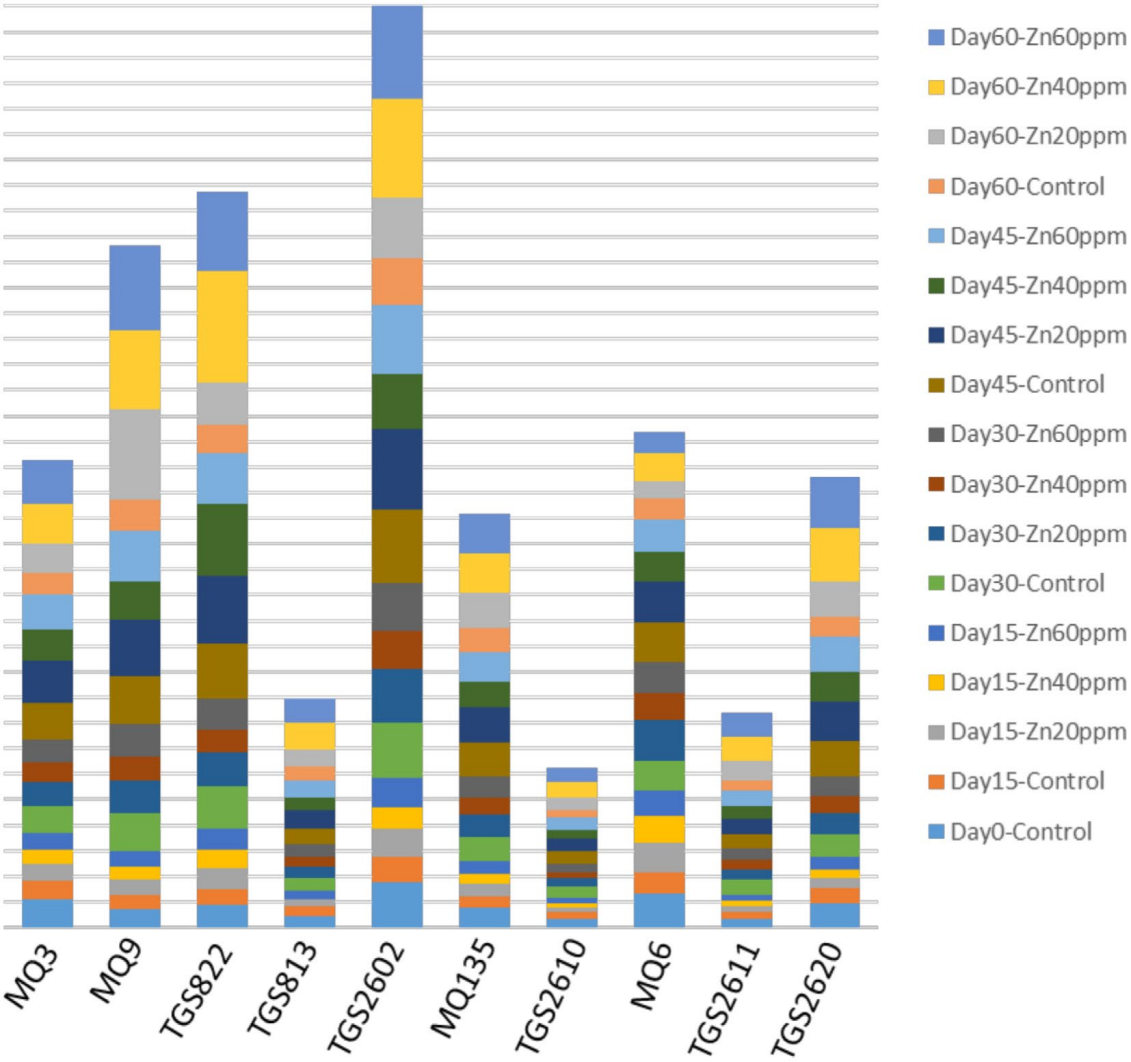
Changes in sensor responses for each sample during storage.

As shown in Figure 6, the sensors TGS822, TGS2602, and MQ9 exhibited the most robust responses to the aromatic compounds, consistent with the PCA loading plot results. Additionally, Figure 5 illustrates no significant differences in the aromatic compounds between different zinc concentrations at specific storage periods. Therefore, it can be concluded that zinc fortification had no significant impact on the aromatic profile, which can have a positive effect on consumer choice. Because customers prefer to use paste with a natural smell and the lack of significant difference between the smell of the fortified tomato paste and the control sample can provide nutrients without negatively affecting the customer's choice. However, significant differences in the aromatic compounds were observed across different storage periods for the same zinc concentration, aligning with the PCA results. This variation may be due to the concentration of the samples or the increase in the concentration of aromatic compounds within the enclosed sample container.

**FIGURE 7:**
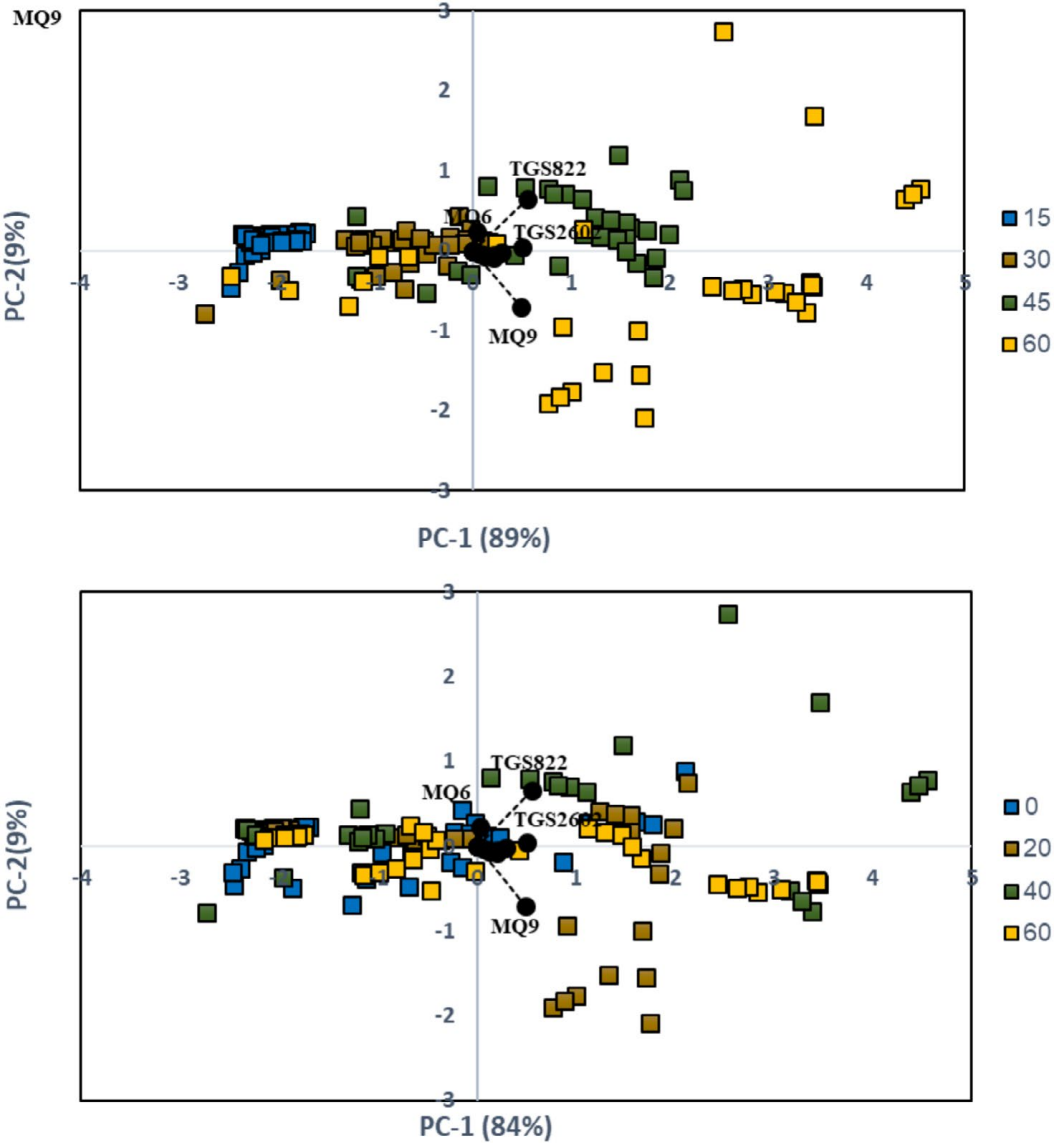
PCA results for samples with different storage periods and zinc concentrations.

Furthermore, the aromatic compounds enabled the classification and differentiation of the samples based on zinc concentration (0, 20, 40, and 60 ppm) and storage period (15, 30, 45, and 60 days). The PCA results are presented in Figure 7, while the confusion matrix results from the QDA method are shown in Tables 3 and 4. The score plot of the electronic nose data for the four storage periods (15, 30, 45, and 60 days) (Figure 7) clearly distinguished the samples based on storage time. PC‐1 and PC‐2 accounted for 89% and 9% of the variance in the input data, respectively. Based on the loading plot values, it can be concluded that all sensors positively contributed to PC‐1, with TGS822, TGS2602, and MQ9 having the most substantial positive impact. TGS822 and MQ9 showed the most significant positive and negative effects on PC‐2. The score plot for the four zinc concentrations (0, 20, 40, and 60 ppm) revealed substantial overlap among the groups, indicating no clear distinction between them. However, PC‐1 and PC‐2 explained 84% and 9% of the variance, respectively. The loading plot indicated that the sensors had similar effects on PC‐1 and PC‐2 across various storage periods. Based on the score and loading plots, it can be concluded that PCA effectively distinguished samples by storage periods, demonstrating the significant impact of storage time on the release of aromatic compounds. However, the method was less effective at differentiating samples with varying zinc concentrations due to overlapping scent profiles, suggesting that zinc levels did not significantly affect the aromatic compounds. Similar results were found in a study by Gholami et al. which used an electronic nose to examine the effects of storage periods and packaging conditions on mushrooms. Their study employed PCA to differentiate samples by storage periods, temperatures, packaging films, and gases. The results showed distinct separations for different temperatures, packaging films, and gases, but overlap occurred between storage periods (Gholami et al. 2023). Likewise, Feng et al. used PCA to classify tomato quality with an electronic nose under different argon pressures during a short storage period (Feng et al. 2018). Their findings confirmed that PCA was an effective tool for distinguishing between samples. Additionally, Gomez et al. successfully applied PCA to classify tomatoes based on storage time (Gómez et al. 2008).

**TABLE 3:** Confusion matrix for classifying zinc concentrations in tomato paste.

	0	20	40	60	Sensivity	Specificity	Accuracy
0	29	0	0	0	0.91	1.00	0.97
20	3	29	0	1	0.91	0.96	0.94
40	0	0	26	0	0.81	1.00	0.95
60	0	3	6	31	0.97	0.90	0.92

**TABLE 4:** Confusion matrix for classifying storage periods in tomato paste samples.

	15	30	45	60	Sensivity	Specificity	Accuracy
15	32	0	0	0	1.00	1.00	1.00
30	0	32	0	0	1.00	1.00	1.00
45	0	0	32	0	1.00	1.00	1.00
60	0	0	0	32	1.00	1.00	1.00

The confusion matrix for classifying samples by zinc concentration in tomato paste using the QDA method is presented in Table 3. This method accurately classified 26, 29, 29, and 31 out of 32 samples for zinc concentrations of 0, 20, 40, and 60 ppm, respectively. The highest accuracy (97%) was achieved in distinguishing samples containing 60 ppm of zinc. The overall accuracy of the method in classifying the samples into four groups (control, fortified with 20, 40, and 60 ppm zinc) was 90%. Table 4 presents the confusion matrix for classifying the tomato paste samples by storage period. The overall accuracy of the method in classifying the samples into four categories (15, 30, 45, and 60 days) was 100%, with all 32 samples correctly classified and no misclassifications. These results suggest that the samples with different storage periods had more distinct aromatic profiles than those with varying zinc concentrations, corroborating the PCA findings. In another study, the shelf life of edible oil samples across different storage periods was classified using an electronic nose and the QDA method, achieving a classification accuracy of 95% (Karami et al. 2021). In another study, the QDA method was used to classify Golab apples based on storage time, with an accuracy of 83.33% (Lashgari and MohammadiGol 2016). Mahmodi et al. also successfully applied the QDA method to classify diesel‐biodiesel blends (Mahmodi et al. 2019).

## 4 Conclusion

Fortifying tomato paste with zinc can be an effective strategy to enhance its nutritional value and significantly improve its quality preservation and stability during storage. In this study, industrial tomato paste was fortified with zinc at 20, 40, and 60 ppm concentrations, and the samples were stored at 4°C for 60 days. The results showed a general decline in product quality over time. However, zinc fortification helped mitigate changes in tomato paste's physical, chemical, and microbiological properties during storage. The zinc‐fortified samples experienced the least variation in all measured characteristics. Regarding the electronic nose system data, the score plot for the four zinc concentrations (0, 20, 40, and 60 ppm) showed significant overlap among the groups, with no distinct pattern to separate them. However, PC‐1 and PC‐2 accounted for 84% and 9% of the variance in the input data, respectively. The QDA method successfully classified 26, 29, 29, and 31 out of 32 samples for zinc concentrations of 0, 20, 40, and 60 ppm, respectively, with the highest classification accuracy of 97% for samples containing 60 ppm zinc. The electronic nose system showed moderate effectiveness in distinguishing samples based on zinc concentration, with the best classification accuracy observed for the highest zinc concentration (60 ppm).

## Author Contributions

**Rashid Gholami:** conceptualization (equal), data curation (equal), formal analysis (equal), funding acquisition (equal), investigation (equal), project administration (equal), resources (equal), software (equal), supervision (equal), validation (equal), visualization (equal), writing – original draft (equal), writing – review and editing (equal). **Nahid Aghilinategh:** conceptualization (equal), methodology (equal), software (equal), validation (equal), writing – original draft (equal), writing – review and editing (equal).

## Consent

The authors have nothing to report.

## Conflicts of Interest

The authors declare no conflicts of interest.

## Data Availability

The data that support the findings of this study are available on request from the corresponding author.
